# Development of an *ex Vivo* Method for Multi-unit Recording of Microbiota-Colonic-Neural Signaling in Real Time

**DOI:** 10.3389/fnins.2018.00112

**Published:** 2018-02-27

**Authors:** Maria M. Buckley, Dervla O'Malley

**Affiliations:** ^1^Department of Physiology, University College Cork, Cork, Ireland; ^2^APC Microbiome Institute, University College Cork, Cork, Ireland

**Keywords:** vagus, microbiota-gut-brain axis, extracellular electrophysiology, microdissection, distal colon

## Abstract

**Background and Objectives:** Bidirectional signaling between the gastrointestinal tract and the brain is vital for maintaining whole-body homeostasis. Moreover, emerging evidence implicates vagal afferent signaling in the modulation of host physiology by microbes, which are most abundant in the colon. This study aims to optimize and advance dissection and recording techniques to facilitate real-time recordings of afferent neural signals originating in the distal colon.

**New Protocol:** This paper describes a dissection technique, which facilitates extracellular electrophysiological recordings from visceral pelvic, spinal and vagal afferent neurons in response to stimulation of the distal colon.

**Examples of Application:** Focal application of 75 mM KCl to a section of distal colon with exposed submucosal or myenteric nerve cell bodies and sensory nerve endings evoked activity in the superior mesenteric plexus and the vagal nerve. Noradrenaline stimulated nerve activity in the superior mesenteric plexus, whereas application of carbachol stimulated vagal nerve activity. Exposure of an *ex vivo* section of distal colon with an intact colonic mucosa to peptidoglycan, but not lipopolysaccharide, evoked vagal nerve firing.

**Discussion:** Previous studies have recorded vagal signaling evoked by bacteria in the small intestine. The technical advances of this dissection and recording technique facilitates recording of afferent nerve signals evoked in extrinsic sensory pathways by neuromodulatory reagents applied to the distal colon. Moreover, we have demonstrated vagal afferent activation evoked by bacterial products applied to the distal colonic mucosa. This protocol may contribute to our understanding of functional bowel disorders where gut-brain communication is dysfunctional, and facilitate real-time interrogation of microbiota-gut-brain signaling.

## Background and objectives

Communication between the gastrointestinal (GI) tract and the brain, or the “gut-brain axis” utilizes endocrine (Gareau et al., 2008; Rhee et al., 2009) and immune mediators (Öhman et al., 2015) and peripheral afferent and efferent nerves. Sympathetic and parasympathetic efferent nerves synapse with myenteric and submucosal neurons and innervate the submucosal and muscle layers, facilitating regulation of GI function (Koeppen and Stanton, 2009; Powley et al., 2011; Boron and Boulpaep, 2016). The sensory arm of the bidirectional gut-brain axis consists of extrinsic sensory neurons (vagal, pelvic, and thoracolumbar spinal afferent neurons). Vagal fibers innervate myenteric neurons, smooth muscle and mucosal layers, where they are well-positioned to sense chemonociceptive signals (Wang and Powley, 2000; Lamb et al., 2003; Powley et al., 2011). However, they are not thought to reach through the epithelium into the gut lumen. Mechanosensory spinal afferents terminate in the serosa, muscularis externa, myenteric and submucosal plexi, mucosa and around extramural and intramural blood vessels as well as collaterals in prevertebral (sympathetic) ganglia, and many visceral afferents are polymodal, responding to more than one stimulus modality (Su and Gebhart, 1998; Brierley et al., 2004). Vagal nerve cell bodies are found in the nodose ganglion and central terminals of the nucleus tractus solitarius in the brainstem. Spinal visceral afferent nerves have cell bodies in the dorsal root ganglia and central terminals of the superficial dorsal horn of the spinal cord. The vagus nerve is generally thought to innervate the upper intestinal tract, ascending and transverse sections of the colon (Buijs and Swabb, 2013) however, vagal afferents supplying the rat distal colon have also been identified in lipophillic tracer studies (Berthoud et al., 1997; Herrity et al., 2014). Moreover, a number of studies (Lowry et al., 1951; Tong et al., 2010; Herrity et al., 2014; Howland, 2014), including those investigating the capacity of commensal probiotic strains to modify centrally-regulated behaviors (Bercik et al., 2011; Bravo et al., 2011), support the existence of vagally-mediated signaling arising from the colon. This may be due to direct activation of colonic afferents or possibly via intrinsic primary afferent neurons (IPANs) (Perez-Burgos et al., 2014), which are located within the submucosal and myenteric plexi.

Recent focus on the capacity of luminal microbial and liver factors to signal to the central nervous system (CNS) (Bercik, 2011; Di Mauro et al., 2013; Schroeder and Bäckhed, 2016) has resulted in re-naming of the signaling pathway as the “microbiota-gut-brain axis.” There are trillions of microbes, primarily bacteria, in the human gut with the highest density in the colon. The importance of luminal microbiota in the normal development of the immune, endocrine and nervous systems has been established using germ-free mice, and absence of microbiota causes changes in centrally-mediated behaviors (Clarke et al., 2014). The role of microbes in a neurally-mediated signaling pathway was elegantly demonstrated using electrophysiological studies which showed that IPANs are less excitable in germ-free mice (McVey Neufeld et al., 2015).

New methodological techniques such as high-throughput DNA sequencing, bioinformatics and gnotobiotic animal models have emerged in recent years (Blaser, 2014), resulting in large strides in our understanding of the microbial-host dialogue. The importance of the vagus in microbiota-gut-brain signaling (Bercik et al., 2011; Bravo et al., 2011), suggests the existence of a neural signaling mechanism from the distal gut, where the density of microbes is highest. However, our understanding of the cellular and molecular mechanisms of how colonic microbial moieties signal in real time to the CNS has been hampered by limitations in our physiological research techniques.

Recent studies have used electrophysiological techniques to record changes in activity in vagal afferents in the jejunum following stimulation with commensal bacterial strains (Perez-Burgos et al., 2013, 2015). Moreover, real-time recording of lumbar splanchnic and pelvic nerve activity in response to stimuli in the distal colon have been described (Brierley et al., 2004). The objectives of this study were to record afferent neurons in response to pharmacological stimulation of the distal colon. This technical development facilitates multi-unit extracellular recordings of real-time afferent signals arising in the distal colon and screening of microbial factors with neuro-modulatory actions. It may also be applied to determine the molecular and cellular mechanisms underlying interactions between microbes and their mammalian hosts.

## Protocol

### Ethical approval

This study was carried out under national license in full accordance with European Union directives with the recommendation of the institutional (University College Cork Animal Experimentation Ethics Committee) animal ethics committee. The protocol was approved by University College Cork Animal Experimentation Ethics Committee.

### Animals

Male Sprague Dawley (SD) rats (8–12 weeks, 200–300 g) were bred in the Biological Services Unit, University College Cork, and group housed 4 per cage and maintained on a 12/12-h dark-light cycle (08.00–20.00) with a room temperature of 22 ± 1°C. Rats had access to food and water *ad libitum*. Animals were sacrificed by CO_2_ overdose and exsanguination.

### Dissection protocol

#### Preparation of the vagal and mesenteric afferents

**Figure 1:**
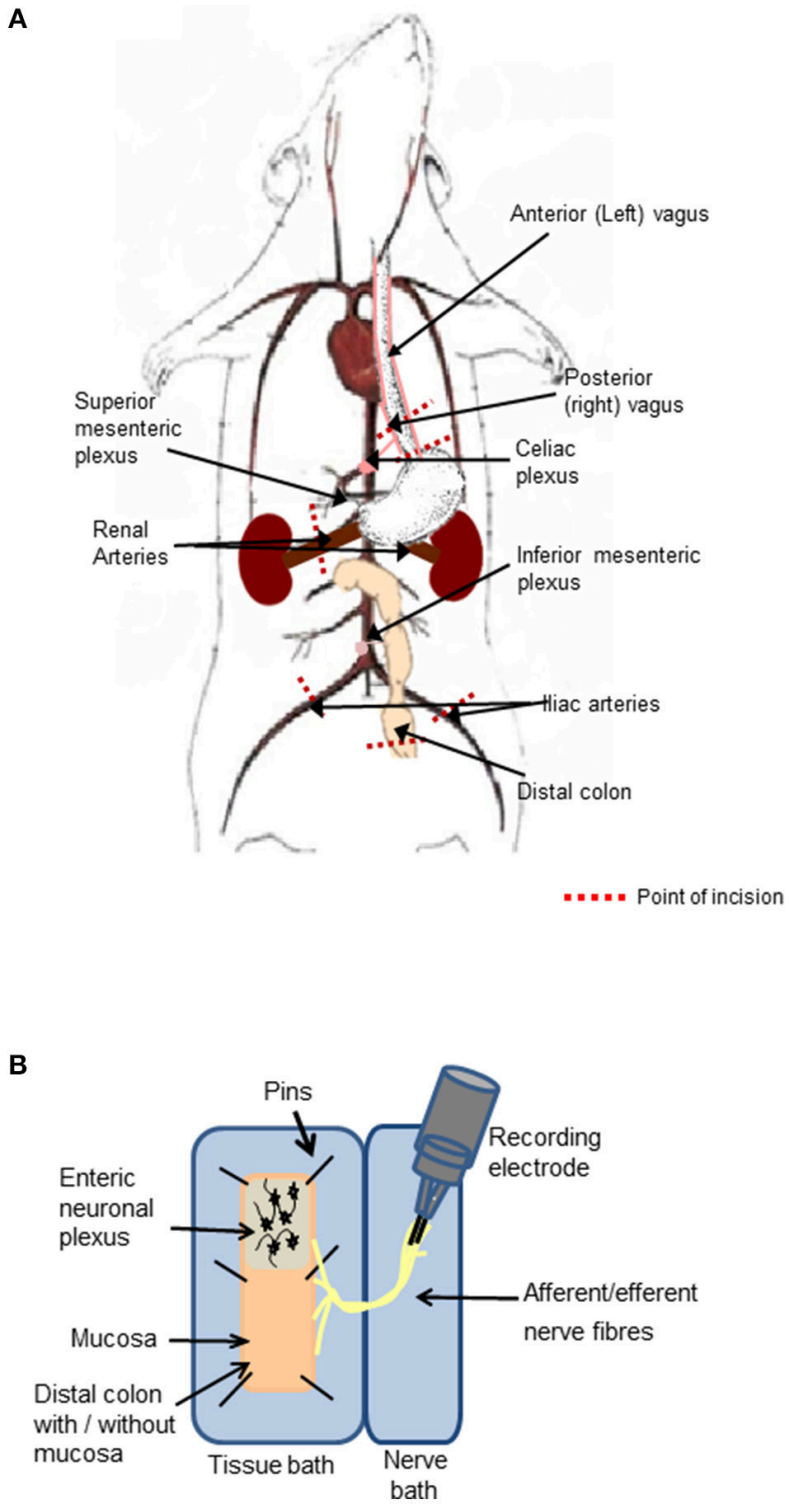
Preparation of the distal colon with intact peripheral innervation. **(A)** The animation illustrates anatomical markers of where to excise the vagal nerve with intact distal colon. Cut lines are indicated by red dashed lines. **(B)** The *ex-vivo* tissue preparation incorporating the distal colon with mucosa either intact or removed to expose the enteric neurons and associated afferent endings pinned out in a Krebs saline-filled tissue bath and the intact peripheral nerve bundles fed through to the adjacent Krebs saline-filled nerve recording chamber, where neural activity was recorded on bipolar recording electrodes.

A vertical abdominal incision was made below the sternum to expose the small and large intestine with intact peripheral nerves. The diaphragm was separated from the sternum and ribs, and an incision made through the diaphragm to the esophagus. The right and left branches of the vagus nerve were apparent on the posterior and anterior aspects of the esophagus, respectively. A section of the esophagus was excised (between 1 and 3 mm superior to the stomach), while ensuring the posterior (right) vagus and its branches were kept intact. The celiac branch of the vagus was dissected to the level of the celiac ganglia, which is located posterior to the stomach, anterior to the crus of the diaphragm and superior to the superior mesenteric plexus corresponding to the first lumber vertebra (Figure 1A).

#### Preparation of the isolated distal colon with attached neurovascular bundle

A 5 cm section of the distal colon was excised (~6 cm from the anal sphincter) taking care to maintain the innervation through the mesentary. The iliac arteries and veins were sectioned and tied off 1 cm inferior of the bifurcation of the abdominal aorta and inferior vena cava, to allow for excision of the abdominal aorta and inferior vena cava with intact mesenteric ganglia. The inferior mesenteric plexus, abdominal aorta and inferior vena cava were precisely excised at a level corresponding to the renal vessels. The renal vessels were sectioned and tied off 1 cm to the right and left of the right and left aorticorenal ganglia, respectively and the excision continued to 1 cm superior to the celiac plexus (Figure 1A).

The final tissue preparation included the distal colon, which may be opened out to expose the luminal mucosa or the mucosa may be removed to expose the underlying intrinsic neuronal networks and extrinsic nerve endings. The colon was opened longitudinally, off center to the mesentery border to orientate the intact nerve fibers to the edge of the opened tissue. The nerve fibers arising from the distal colon extended to the inferior and superior mesenteric ganglia, celiac ganglia, and to the vagus nerve, which remained attached to the esophagus.

#### Experimental and recording chambers

The nerve-gut recording rig consisted of two aligned Sylgard- (Sylgard 184 silicone elastomer kit, WPI, Sarasota FL, USA) lined polymethyl methacrylate (perspex) chambers. The chamber housing the colon (2.5 × 10 cm) was separated from the adjacent nerve chamber (1.5 × 10 cm, Figure 1B) by a 2 mm perspex barrier with an opening, through which the nerve fibers were threaded and the gap was sealed with petroleum jelly. To prevent damaging the neuronal cells, glass rods were used to manipulate the nerves and ganglia. Both chambers were superfused with 5% CO_2_/95% O_2_ bubbled Krebs saline comprised of (in mmol l^−1^): NaCl, 117; KCl, 4.8; CaCl_2_, 2.5; MgCl_2_, 1.2; NaHCO_3_, 25; NaH_2_PO_4_, 1.2 and D-glucose, 11, maintained at 37°C. Nifedipine (1 μM) an L-type calcium channel blocker, was present in all studies to suppress smooth muscle activity. The opened colon was pinned out in the dish with the mucosal side facing upwards with continuous saline flow. The serosal side of the tissue was also continuously irrigated from below with 5% CO_2_/95% O_2_ bubbled Krebs saline through perforated plastic tubing embedded in Sylgard. To expose the submucosal neurons, the mucosal layer was stripped away using fine dissection forceps. To expose myenteric neurons, the mucosa and submucosal layers were removed and the circular muscle fibers were peeled away using fine dissection forceps leaving a distal colonic longitudinal muscle myenteric plexus (LMMP) preparation and extrinsic nerve fiber endings. The distal colonic section was exposed to focally applied reagents. The studies were reproduced in at least three separate tissue preparations. The order in which drugs were applied was randomized to ensure that the responses were reproducible, and no form of tachyphylaxis contributed to the neural response evoked.

#### Electrophysiological recordings from neural ganglia or nerve fibers

In the Kreb's saline-filled nerve recording chamber the superior mesenteric ganglia and teased apart vagal nerve bundles were covered with liquid paraffin. Neural activity was recorded using platinum bipolar recording electrodes (WPI, Sarasota, FL, USA) and data acquisition hardware (Power lab, AD Instruments, Oxford, UK). Paired recordings of nerve activity were taken from the superior mesenteric plexus and celiac branch of the vagus concurrently. Extracellular recording signals were amplified using a BioAmp amplifier (AD Instruments), which has a high impedence and was electrically isolated with a mains filter. High- and low-pass filters were set at 0.2 and 2 KHz. The signal was sampled every 10 ms, digitized to enable generation of integrated nerve activity and stored on a hard drive for later offline analysis.

On completion of the experiment, background noise was recorded for a period of 15 min and subsequently subtracted from the raw data to enable analysis of the filtered responses. Traces reflect compound neural activity from multiple neurons within the superior mesenteric ganglia and multiple axons in the vagus nerve. The nerve activity was viewed and analyzed with Chart 7 (AD Instruments). Statistical analysis was carried out on the differences in area under the curve calculated from the filtered traces.

### Statistical analyses

Statistical analysis was carried out using GraphPad prism for Windows (version 5). Changes in multi-unit neural activity in the superior mesenteric ganglia and vagal nerve are presented as area under the curve (AUC), from rectified traces. The data are represented as mean values ± the standard error of the mean. Paired *t*-test was used and *p* ≤ 0.05 was considered significant.

### Examples of application of colonic nerve recording

#### Neural stimulation in the distal colon evoked nerve activity in the superior mesenteric plexus

The described dissection and recording technique facilitates real-time multi-unit electrophysiological recording of afferent neural signals originating in the distal colon sent to the superior mesenteric ganglia and the vagus nerve. This is an advancement on previously reported methods (Berthoud et al., 2001; Brierley et al., 2004; Perez-Burgos et al., 2013, 2015). Validation of the technique and examples of the results generated using this protocol are described below.

**Figure 2:**
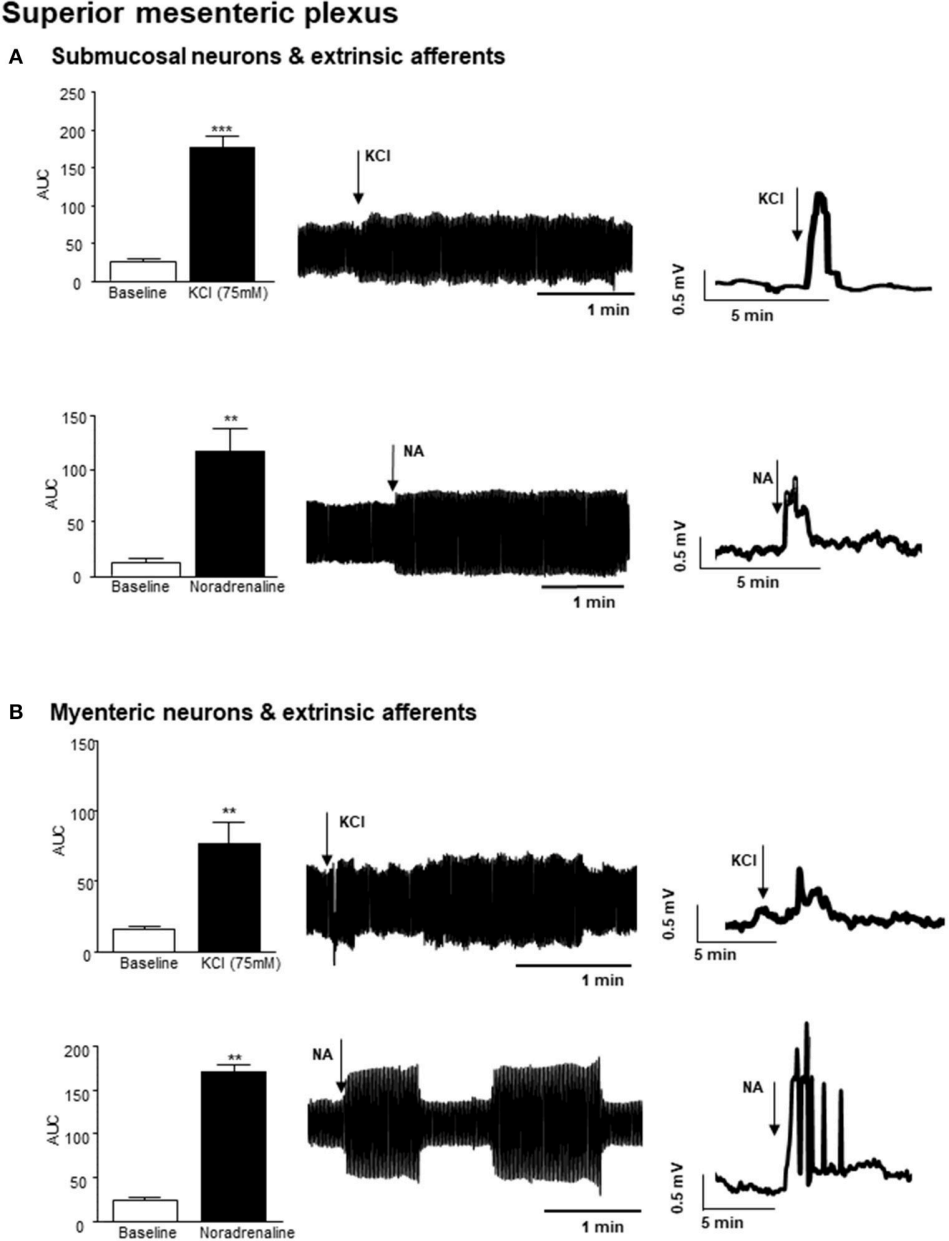
Nerve activity in the superior mesenteric plexus evoked by stimulation of the distal colon. **(A)** The bar charts and representative filtered and unfiltered traces illustrate nerve activity recorded from the superior mesenteric plexus in response to application of potassium chloride (KCl, 75 mM, *n* = 3) and noradrenaline (NA, 8 μM, *n* = 4) to submucosal neurons and afferent nerves in the distal colon. **(B)** The bar charts and representative filtered and unfiltered traces illustrate nerve activity at the superior mesenteric plexus in response to application of KCl (75 mM, *n* = 3) and noradrenaline (NA, 8 μM, *n* = 4) to longitudinal muscle myenteric plexus tissue from the distal colon. ^**^p < 0.01 and ^***^p < 0.001, respectively.

Small bursts of nerve firing were evident in simultaneous recordings from both the superior mesenteric plexus and the vagus nerve innervating a section of distal colon, where the mucosa has been removed to expose sensory endings of vagal and spinal afferents and submucosal neurons, which are important regulators of absorption and secretion. However, direct application of a depolarizing stimulus (75 mM KCl, 3 min) to the colonic tissue, elicited a rapid but transient increase in nerve activity at the superior mesenteric ganglia (*n* = 3 colonic-nerve tissue preparations, *p* < 0.001, Figure 2A). Application of noradrenaline (8 μM, 3 min), the primary neurotransmitter used by the sympathetic nervous system, to submucosal neurons and extrinsic sensory axon terminals also increased neural activity at the superior mesenteric ganglia (*n* = 3, *p* < 0.01, Figure 2A).

Focal application of 75 mM KCl (3 min) to a distal colonic LMMP tissue preparation, similarly resulted in a robust but transient increase in nerve activity in the superior mesenteric plexus (*n* = 3, *p* < 0.01, Figure 2B). Furthermore, application of noradrenaline (8 μM, 3 min) to this tissue preparation resulted in a robust increase in neural activity in the superior mesenteric ganglion (*n* = 3, *p* < 0.01, Figure 2B).

#### Neural stimulation in the distal colon evoked vagal nerve activity

**Figure 3:**
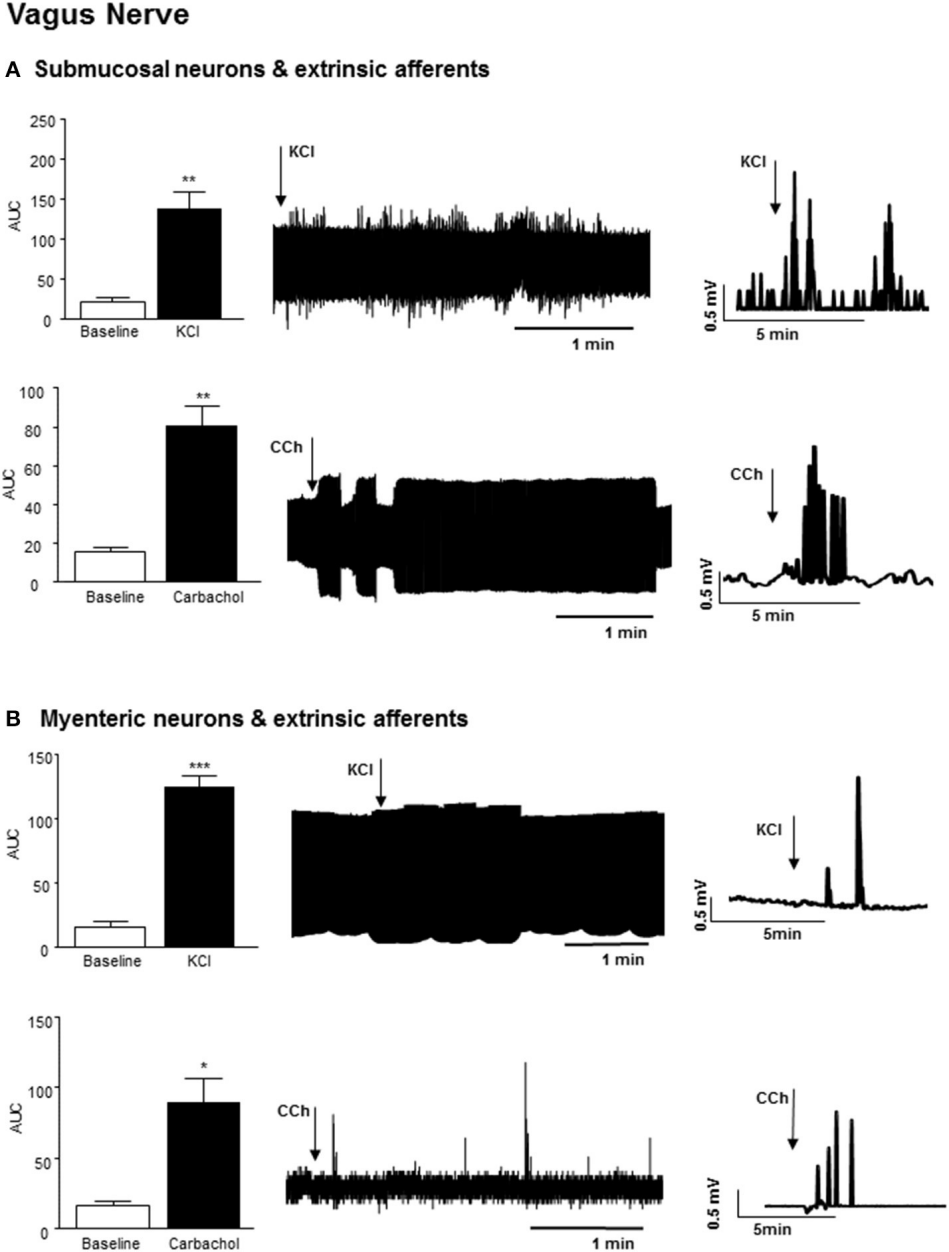
Vagal nerve activity evoked by neural stimulation in the distal colon. **(A)** The bar charts and sample filtered and unfiltered traces show increased nerve firing in the vagus nerve in response to exposure of submucosal neurons and the extrinsic afferents of the distal colon to KCl (75 mM, *n* = 3) and carbachol (CCh, 100 μM, *n* = 3). **(B)** The bar charts and representative traces shows increased vagal nerve firing in response to application of KCl (75 mM, *n* = 3) and carbachol (CCh, *n* = 3) to longitudinal muscle myenteric plexus preparation from the distal colon. ^*^p < 0.05, ^**^p < 0.01, and ^***^p < 0.001, respectively.

Application of 75 mM KCl (3 min) to a section of distal colon with exposed submucosal neurons and sensory afferents also elicited an increase in vagal nerve firing (*n* = 3, *p* < 0.01), which persisted after the stimulus was removed (Figure 3A). Exposure of this distal colonic tissue preparation to an acetylcholine receptor agonist, carbachol (100 μM, 3 min) also immediately increased the frequency of nerve firing in the vagus nerve (*n* = 3, *p* < 0.01, Figure 3A).

Exposure of colonic LMMP tissue to 75 mM KCl (3 min) evoked a robust increase in vagal nerve activity (*n* = 3, *p* < 0.001, Figure 3B). Focal application of carbachol (100 μM, 3 min) to the LMMP colonic tissue similarly evoked a rapid but transient increase in vagal firing (*n* = 3, *p* < 0.05, Figure 3B).

#### Stimulation of colonic mucosa evoked vagal nerve activity

**Figure 4:**
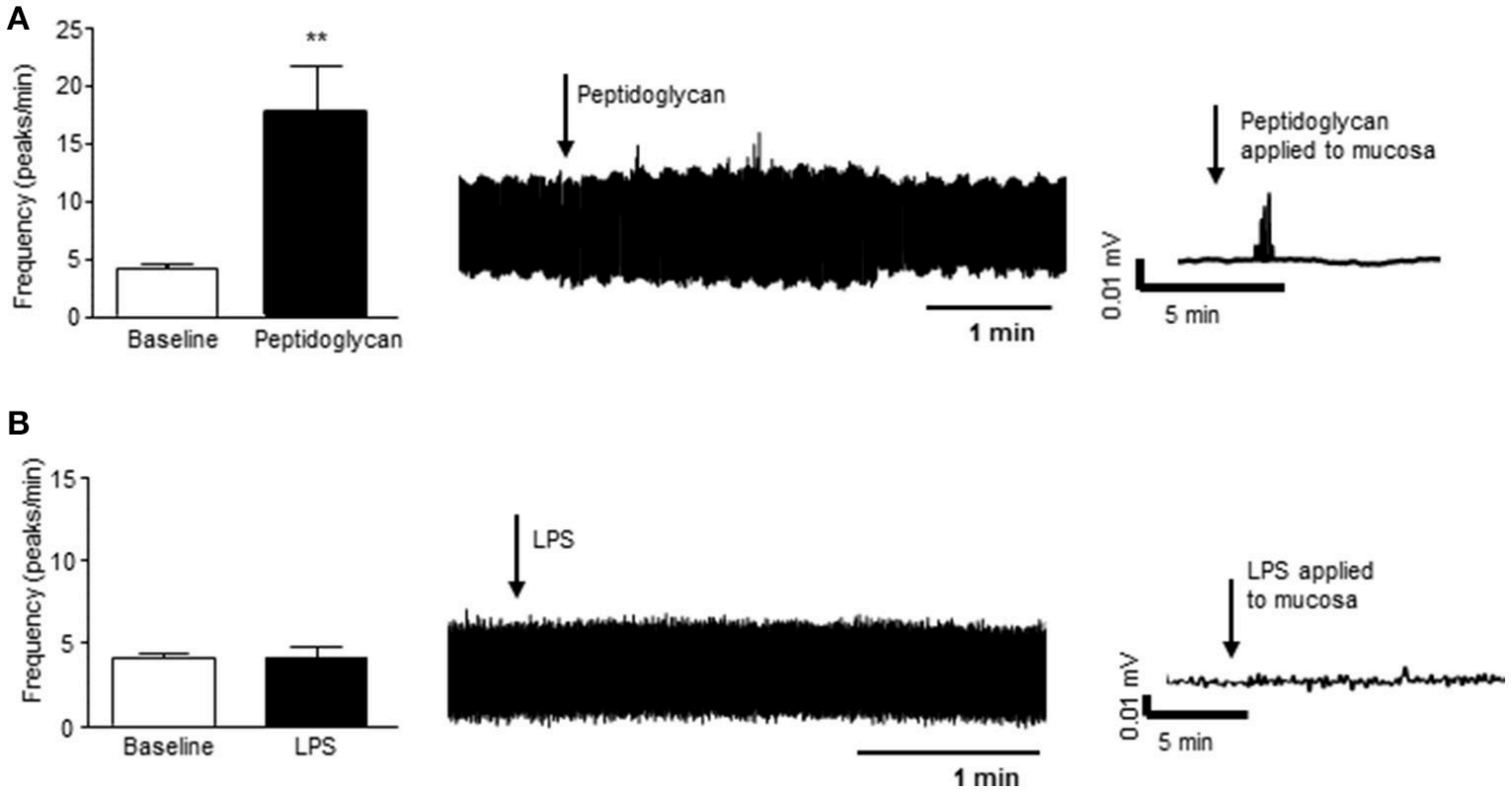
Gram positive bacterial product stimulates vagal firing. **(A)** The bar chart and representative vagal nerve traces illustrate that when peptidoglycan (1 μg/ml, *n* = 4), a gram positive bacterial coat protein, is applied to the mucosa of the distal colon, vagal nerve activity is increased. **(B)** In contrast, lipopolysaccharide (LPS, 2 μg/ml, *n* = 4), the gram negative bacterial coat protein, has no effect on vagal nerve activity. ^**^p < 0.01.

In a segment of distal colon with intact mucosa, application of peptidoglycan (1 μg/ml, 3 min), a major component of the wall of gram positive bacteria and a ligand for toll like receptor (TLR) 2, resulted in increased nerve firing in the vagus (*n* = 4, *p* < 0.01, Figure 4A). Lipopolysaccharide (LPS, 2 μg/ml, 3 min), the external coating of gram negative bacteria and a ligand for TLR4, had no effect on vagal nerve activity (*n* = 4, *p* > 0.05, Figure 4B).

## Discussion

The described dissection and recording technique represents an advancement on previous methods of electrophysiologically interrogating gut-to-brain signaling (Berthoud et al., 2001; Brierley et al., 2004; Perez-Burgos et al., 2013, 2015). This technique facilitates real-time recording of neural afferent signals originating in the distal colon. Given the importance of the bidirectional signaling pathways between the gut and brain both in the physiological regulation of digestion and excretion and also in disorders caused by brain-gut dysfunction, such as functional bowel disorders, this technique may be of great value in understanding the cellular and molecular events underlying these processes. Although limitations of this *ex vivo* technique include a lack of a functioning circulation and possible damage to the epithelial barrier, this method has the potential to screen bacterial products for neuromodulatory activity and allow investigation of cellular and molecular mechanisms of microbe-host communication.

It was previously generally accepted that the vagus nerve innervates the proximal and transverse colon but that the distal colon is exclusively innervated by sacral nerve fibers (Berthoud et al., 1990). Indeed, human anatomy textbooks report vagal innervation extends to the transverse colon but terminates at the left colic flexure (Standring, 2016). However, sympathetic and vagal fibers become “indistinguishably mixed” upon entry to the abdomen (Shoja et al., 2013) and the exact innervation of the distal colon is ambiguous. Retrograde tracers provide evidence that there are vagal afferents with sensory endings in the colon and cell bodies in the nodose ganglion (Herrity et al., 2014), leading to the suggestion that the vagus nerve is substantially involved in lower gastrointestinal function. Our findings support this assertion, as exposure of enteric neurons and afferent neuronal endings of the distal colon to neuromodulatory reagents evoke a rapid increase in activity in neural afferents. 75 mM KCl, which induces neuronal depolarization, caused increased afferent nerve activity in both the superior mesenteric plexus and the vagus nerve. Seventy-five millimolar KCl induced similar results when applied to a section of distal colon with exposed submucosal neurons and extrinsic afferents terminating in this layer of the colon or exposed myenteric neurons with associated extrinsic afferent endings. There is some circumstantial evidence that nutrient-associated release of enteroendocrine hormones causes activation of ENS signaling, which in turn, activates vagal afferents which terminate in the dorsal vagal complex. CCK of neural origin was implicated in their studies and the authors suggest that this may have been released from the enteric neurons (Ritter et al., 1994). Moreover, the trypsin inhibitor, camostat, reduced food intake and increased c-Fos expression in the dorsal vagal complex and submucosal neurons via CCK. Subdiaphragmatic vagotomy abolished c-Fos expression in the dorsal motor complex but also significantly reduced cFos expression in submucosal neurones (Raboin et al., 2008). Additionally, Perez-Burgos and colleagues have provided evidence that a commensal bacterial strain can activate vagal firing using an IPANs as intermediaries (Perez-Burgos et al., 2014). Nonetheless, direct stimulation of extrinsic afferents is the most likely route of vagal nerve and superior mesenteric plexus activation. An alternative possibility is activation of viscerofugal neurons, which may link enteric circuits with the sympathetic nervous system (Sharkey et al., 1998) and can be activated by cholinergic agonists (Hibberd et al., 2012).

Application of noradrenaline to the colonic tissue preparation, with either submucosal or myenteric neurons and their associated afferents exposed, evoked increased nerve activity in the superior mesenteric plexus. Carbachol, the cholinergic agonist, evoked increased activity in the vagus nerve when applied to either tissue preparation, effects most likely to be mediated via direct neuronal activation of extrinsic afferents. Indeed, noradrenaline has been shown to activate sensory nerves (Jørum et al., 2007). Direct activation of smooth muscle or indirect activation via myenteric neurons could also stimulate visceral afferents. However, inclusion of the L-type calcium channel blocker, nifedipine in all solutions to inhibit smooth muscle contraction tends to make this possibility less likely.

Finally, we have demonstrated that a gram positive but not a gram negative bacterial wall component stimulates vagal afferent firing when applied to the mucosa. Given that afferent fiber endings are not thought to penetrate through the epithelial layer, this finding suggests that cellular or molecular intermediaries translate the bacterial signal across the gut barrier to the host peripheral nervous system.

This dissection and recording technique of the distal colon with intact nerve bundles is likely to aid in our understanding of the pathophysiology of functional bowel disorders, where brain-gut communication is disrupted. Perhaps most excitingly, this technique may be used to investigate cellular and molecular mechanisms by which moieties in the external environment of the colonic lumen, including bacteria, phages, fungi and digestive and liver products, can signal to the host nervous system across the gut barrier leading to altered behaviors. These include anxiety and changes in sociability in addition to changes in physiological function, such as neuroinflammation, activation of the stress response, neurotransmission, and neurogenesis (Sherwin et al., 2017). Given the importance of the vagus in bacterial signaling from the gut lumen to the CNS (Bercik et al., 2011; Bravo et al., 2011; Malick et al., 2015), this technique together with pharmacological reagents has the potential to help clarify how microbes are communicating with their host, thus revealing mechanisms underlying the beneficial and detrimental effects of colonic microbes.

## Author contributions

MB: Designed the study, analyzed the data and drafted the manuscript; DO: Designed the study and critically reviewed the manuscript.

### Conflict of interest statement

The authors declare that the research was conducted in the absence of any commercial or financial relationships that could be construed as a potential conflict of interest.
